# Distribution of 37 human papillomavirus types in parotid gland tumor tissues

**DOI:** 10.3892/ol.2013.1770

**Published:** 2013-12-23

**Authors:** WEI-QIANG TENG, XIAO-PING CHEN, XIAO-CHENG XUE, YI ZHANG, XUE-JUN TAN, GUANGBIN SUN, YAN WANG, LI WANG

**Affiliations:** 1 Graduate College, Ningxia Medical University, Yinchuan, Ningxia 750004, P.R. China; 2 Department of Otolaryngology, Shanghai Pudong New Area Gongli Hospital, Shanghai 200135, P.R. China; 3 Department of Otolaryngology, Wanzhou Shanghai Hospital, Chongqing 404100, P.R. China

**Keywords:** parotid gland tumors, risk factor, human papillomavirus, genotyping, flow-through hybridization

## Abstract

Human papillomavirus (HPV) infection has been shown to be associated with human tumorigenesis. The aim of the present study was to demonstrate the association between HPV infection and parotid gland tumors. Paraffin-embedded tissue sections from 59 cases of parotid gland tumors and 20 normal oral mucosa were subjected to DNA extraction and flow-through hybridization and gene chip technology to detect infection of 37 HPV types. The HPV-positive rate was 57.6% in parotid gland tumor paraffin-embedded tissue specimens, whereas, the normal control group was negative for HPV. The HPV-positive rate was 59.6% in parotid gland benign tumor tissues and 42.9% in parotid malignant tissues. HPV infection in parotid gland tumors was dominated by the high-risk subtypes (80.7%), which mainly consisted of HPV 16, 18 and 52 (61.4%). In addition, parotid gland tumor tissues were found to be infected by multiple or single types of HPV, but were predominantly infected by mixed HPV types. In this study, we found that the occurrence of parotid gland tumor is correlated with HPV infection.

## Introduction

Salivary gland tumors form in the tissues of salivary glands. The incidence of these tumors varies worldwide and is between 0.4–13.5/100,000 individuals annually, with a malignant tumor rate of ~0.4–2.6/100,000 (1). In Mainland China, salivary gland tumors account for 2.3% of all human tumors and ~20% of oral and maxillofacial tumors. Among these tumors, parotid gland tumors are the most common of salivary gland tumors, followed by submandibular gland minor salivary and sublingual gland tumors. With changes in lifestyle and an increasingly elderly population, the incidence of parotid gland tumors is also increasing. However, the etiology of parotid gland tumors remains to be defined, although, previous studies indicate that there are various risk factors (such as tobacco smoking, alcohol consumption and a previous history of radiotherapy) associated with the development of parotid gland tumors. Previous studies have indicated that mobile phone usage may increase the incidence of parotid gland tumors (2). However, the development of parotid gland tumors, similar to the majority of other tumors, is associated with multiple risk factors. Alteration of various tumor suppressor genes and activation of oncogenes are important in parotid gland tumor development and progression (3–7). However, the underlying molecular mechanisms responsible for their alteration and activation require further investigation. Therefore, the present study focused on the role of human papillomavirus (HPV) infection in the development of parotid gland tumors.

Notably, HPV has previously been shown to induce human types of cancer and it is capable of infecting human keratinocytes and mucous membranes. HPV is a small (45–55-nm in diameter), non-enveloped double-stranded and closed circular DNA tumor virus. HPV infects epithelial tissues through microabrasions or other epithelial trauma by delivery of the viral genome to the host cell nucleus. HPV exists in the following three forms in infected cells: i) Integrated, DNA virus in the host cell chromosome; ii) episomal, DNA virus that is free from the cell chromosome; and iii) episomal and integrated. While the majority of the known types of HPV cause no symptoms in almost all patients, specific types of HPV may cause warts and cancer in humans. For example, HPV E6 and E7 proteins inactivate two tumor suppressor proteins, p53 and pRb. The correlation between HPV and head and neck cancer has been previously well documented in the literature (8–10). Several studies have demonstrated that 50–90% of squamous cell carcinoma of the oropharynx, tongue and tonsils, are associated with HPV infection (11–13). The majority of previous studies have only focused on the role of HPV infection in squamous cell tumors (14,15), however, specific studies have shown that HPV infection may also play a role in glandular epithelial tumors (16,17). Thus, the current study utilized flow-through hybridization and gene chip technology, an analytical technique with high sensitivity and specificity. Specifically, 37 common types of HPV were analyzed in 59 cases of paraffin-embedded specimens of parotid gland tumor tissues to identify the correlation between HPV subtype infection and the development of parotid gland tumors.

## Materials and methods

### Study population

In total, 59 cases of parotid gland tumor tissue samples were obtained from the Department of Pathology, Shanghai Pudong New Area Gongli Hospital (Shanghai, China) between 2004 and 2011. All patients were diagnosed with parotid gland tumors histopathologically and included 35 males and 24 females with an age range between 23 and 89 years (56.7±16.2 years old). Among the 59 cases, 52 were benign tumors (36 mixed tumor, 3 adenoma, 12 gland lymphoma and 1 myoepithelioma) and 7 were malignant tumors (3 adenocarcinoma, 1 adenoid cystic carcinoma, 1 mucoepidermoid carcinoma and 2 other cases). All patients had not previously been treated with any radiotherapy or chemotherapy prior to surgery. Tumor tissue samples were fixed in 10% formalin and embedded in paraffin.

In addition, normal oral mucosa tissues from 20 normal healthy volunteers were also obtained from the hospital and fixed in formalin and embedded in paraffin. Of the healthy volunteers, 50% were male and female, respectively, with an age range between 24 and 62 years (37.9±15.6 years). The study was approved by the hospital review board and each patient and volunteer signed an informed consent form.

### DNA extraction, polymerase chain reaction (PCR) amplification and flow-through hybridization

Genomic DNA was first extracted from each tissue specimen. In brief, paraffin-embedded tissue samples from all 59 cases of parotid gland tumors and 20 healthy volunteers were randomly arranged and three consecutive tissue sections (10-μm thick) were subjected to DNA extraction using a DNA extraction kit (Chaozhou Hybribio Biochemical Co., Ltd., Chaozhou, China) according to the manufacturer’s instructions. Next, the DNA samples were amplified by PCR using a PCR reagent kit (Chaozhou Hybribio Biochemical Co., Ltd.). The two consensus primers, MY09 and MY11, that amplify the most conserved HPV L1 region and have been widely used in previous clinical and epidemiological studies, were used as controls: MY09, 5′-CGTCCMARRGGAWACTGATG-3′; and MY11, 5′-GCMCAGGWCATAAYAATGC-3′. Specifically, PCR amplification contained 1 μl DNA sample in 25 μl PCR mixtures. The PCR conditions were as follows: DNA predenaturation at 95°C for 9 min, followed by 40 cycles of 95°C for 20 sec, 55°C for 30 sec, 72°C for 30 sec and a final extension at 72°C for 5 min. Next, the PCR products were subjected to DNA flow-through hybridization. Briefly, 20 μl PCR product was added to a low-density cDNA microarray containing one of the following 37 HPV type-specific oligonucleotide probes: HPV 6, 11, 16, 18, 26, 31, 33, 34, 35, 39, 40, 42, 43, 44, 45, 51, 52, 53, 54, 55, 56, 57, 58, 59, 61, 66, 67, 68, 69, 70, 71, 72, 73, 82, 83, 84 and CP8304. The manufacturer’s instructions for the HPV GenoArray test kit (Qiagen, Hilden, Germany) were followed for flow-through hybridization and biotin enzyme-color reaction for ≤1 h. Distilled water and HPV 18 were used as the negative and positive controls, respectively.

### Data evaluation

Biotinylated DNA was used as biotin-control points to check the reliability and producibility of the assays utilized. In addition, internal control (IC) spots were used as quality control spots for PCR, thermal denaturation and hybridization processes. If no inhibiting factors were identified in the PCR system, IC spots appear; if one of the two control spots does not appear, the experiments were repeated for that particular specimen. Next, all experimental results were obtained by visual inspection of assayed data. The positive results appeared as clear blue-purple dots. The HPV-positive points were evaluated by matching them to the distribution map of HPV subtypes on the film.

### Statistical analysis

All data were summarized as positive or negative for each case and then statistically analyzed by SPSS 11.5 software (SPSS, Inc., Chicago, IL, USA) and χ^2^ test. P<0.05 was considered to indicated a statistically significant difference.

## Results

### Detection of HPV DNA in parotid gland tumor and normal control tissues

PCR amplification and flow-through hybridization detection of 37 subtypes of HPV were performed in tissue specimens of parotid gland tumors and normal oral mucosa. The results are shown in Tables I and II. Specifically, the total HPV-positive rate in the 59 tissue specimens of parotid gland tumors was 57.6% (34/59), including 59.6% (31/52) in benign parotid gland tumors and 42.9% (3/7) in malignant parotid gland tumors. HPV infection in normal oral mucosa tissue samples was negative. A statistically significant difference was identified in HPV infection between the parotid gland tumor specimens and normal control group (χ^2^=20.234; P<0.05), whereas, the HPV-positive rates between benign and malignant parotid gland tumors were not significantly different (χ^2^=0.189; P>0.05). In the 34 cases of HPV-positive samples, no statistically significant difference was identified in gender distribution (χ^2^=0.008; P=0.928).

### Distribution of HPV types in benign and malignant parotid gland tumor tissues

**Figure 1:**
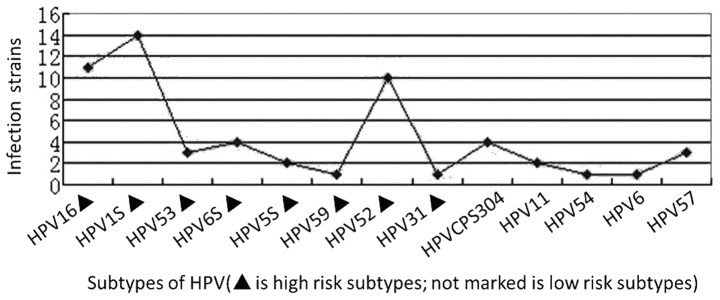
Distribution of high- and low-risk HPV subtypes in parotid gland tumor tissues. HPV, human papillomavirus.

Next, HPV positivity was analyzed in the 34 HPV-positive cases. A total of 57 strains of HPV were identified in these 34 cases, which included 13 subtypes of HPV. The specific distribution of HPV subtypes is shown in Fig. 1. In total, 5 low-risk types, including 11 strains of HPV, represented 19.3% of the total number of detected strains. The predominant subtypes were HPV CP8304 and 57, accounting for 5% of the total number of detected strains. In addition, 46 strains of HPV belonged to 8 high-risk types and accounted for 80.7% of the total number of detected strains. The predominant subtypes were HPV 16, 18 and 52, representing 19.3, 24.6 and 17.5% of the total number of detected strains, respectively. The distribution of HPV subtypes in benign and malignant parotid gland tumors are shown in Tables III and IV, respectively.

### Single and multiple HPV infections in parotid gland tumor tissue specimens

It was determined whether single or multiple HPV infections were present in parotid gland tumor tissue specimens. The results showed that a single or mixed HPV infection by multiple types was detected in specific single tissue samples. The infection rates of single and multiple types were 47.1 (16/34) and 52.9% (18/34), respectively. In the mixed infection, double infections were the most common at 77.8% (14/18), triple infections were 16.7% (3/18) and infections by four types were 5.6% (1/18) (Table V).

## Discussion

In the current study, the presence of infection by various HPV genotypes was determined in 59 cases of parotid gland tumor tissue specimens and compared with 20 cases of normal oral mucosa specimens using a rapid flow-through hybridization of nucleic acid molecular gene chip technology. This technique differs from immunohistochemical staining and PCR due to its high sensitivity and specificity, accuracy and reliability. The HybriMax technique was used to rapidly detect 37 HPV genotypes for mixed HPV infections. The hybridization background was clean and required a small volume of reagents.

In the present study, 57.6% (34/59) of the parotid gland tumor tissue specimens were HPV-positive, whereas, the normal oral mucosa samples were HPV-negative. The results indicated that HPV infection may promote the development of parotid gland tumors. Furthermore, HPV infection rates in benign and malignant parotid gland tumor tissue specimens were 59.6 (31/52) and 42.9% (3/7), respectively, which were not statistically different. HPV 16, 18 and 52 were the predominant HPV types detected in the parotid gland tumor tissue samples. Previous studies have shown that HPV infection rates vary between 0 and 77.8% in parotid gland tumor tissues (8–10,18–21). These significant variations are due to differences in geographical factors, the number of tissue specimens studied and detection methods of HPV subtypes. Previous studies have also shown that parotid gland tumors are predominantly infected by HPV 16 and 18 types (22–25), which is consistent with the results of the current study. The majority of previous studies on HPV investigated cervical diseases and have shown that HPV infection rates increase with the degree of cervical lesions aggravated (26–28). Other studies have shown that increased HPV-positive rates correlate with increments of nasal inverted papilloma pathological classifications and clinical stages (29–31. However, in the current study, no significant differences were identified in HPV-positive rates between benign and malignant parotid gland tumors.

Previous results have demonstrated that infection with mixed or multiple HPV subtypes is associated with the severity of cervical disease (i.e. with an increase in the severity of cervical lesions, multiple infections of HPV occurred). However, subsequent studies have indicated a decline in infections by multiple HPV types and that cervical squamous cell carcinoma is infected by a single high-risk HPV type (32). In addition, infection by multiple HPV types may facilitate the induction of atypical hyperplasia of nasal inverted papilloma (29). The current study showed that parotid gland tumors infected with a single HPV subtype or multiple types were 47.1 and 52.9%, respectively, indicating that parotid gland tumors are more prone to infection by multiple HPV types. However, it remains to be elucidated how HPV infection promotes the development of this type of tumor.

Parotid glands are localized below and in front of the external acoustic meatus within the mandibular fossa and the deep surface of the mandibular ramus. Normally, it is difficult to obtain patient consent to collect normal parotid gland tissues due to its anatomical location. The parotid gland is histoembryologically similar to oral mucosa; thus, in the current study, oral mucosa was used as a normal control. Previous results have shown that infection with high-risk HPV types is a risk factor for oral cancer, the effects of which may surpass that of tobacco smoking in the development of oral cancer (33). The transmission of HPV may occur through oral sex and infect the oral throat and tonsils. The results from a previous meta-analysis (34) of 5,681 patients with head and neck squamous cell carcinoma showed that HPV infection increased the risk of developing head and neck squamous cell carcinoma. In view of the association between the anatomy of the oral cavity and parotid gland, future studies are likely to investigate the correlation between HPV infection and development of oral cancer and parotid gland tumors. HPV-positive oral cancer and parotid gland tumors are likely to be compared with HPV-negative tissue samples for tumor types and gene expression.

Results of the present study indicate that HPV infection is associated with the development of parotid gland tumors, particularly infection by the high-risk HPV subtypes, HPV 16, 18 and 52. Our study demonstrates the association between HPV infection and parotid gland tumors by flow-through hybridization and gene chip.

## Figures and Tables

**Table I:** Expression of HPV in parotid gland tumor and normal control tissues.

		HPV expression		
			
Group	n	Positive, n (%)	Negative, n (%)	P-value
Control	20	0 (0.0)	20 (100.0)	
Parotid gland tumor	59	34 (57.6)	25 (42.4)	0.000

HPV, human papillomavirus.

**Table II:** Expression of HPV in benign and malignant parotid gland tumor tissues.

		HPV expression		
			
Group	n	Positive, n (%)	Negative, n (%)	P-value
Benign tumors	52	31 (59.6)	21 (40.4)	
Malignant tumors	7	3 (42.9)	4 (57.1)	0.664

HPV, human papillomavirus.

**Table III:** Distribution of HPV subtypes in benign parotid gland tumor tissues.

Pathological classification	n	Low-risk HPV subtypes					High-risk HPV subtypes							
	
		11	54	57	6	CP8304	53	68	58	52	16	18	31	59
Mixed tumor	36	1	1	1	1	3	3	3	1	8	8	8	1	0
Adenolymphoma	12	1	0	1	0	0	0	0	0	1	1	2	0	0
Adenoma	3	0	0	1	0	0	0	0	0	1	0	1	0	1
Myoepithelioma	1	0	0	0	0	0	0	0	0	0	0	1	0	0
Total	52	2	1	3	1	3	3	3	1	10	9	12	1	1

In total, 13 subtypes of HPV were detected in benign parotid gland tumors and were mainly HPV 16, 18 and 52. HPV, human papillomavirus.

**Table IV:** Distribution of HPV subtypes in malignant parotid gland tumor tissues.

		Low-risk HPV subtypes	High-risk HPV subtypes			
			
Pathological classification	n	CP8304	68	58	16	18
Adenocarcinoma	3	0	0	0	1	1
Adenoid cystic carcinoma	1	0	0	0	0	0
Mucoepidermoid carcinoma	1	1	0	1	1	0
Others	2	0	1	0	0	1
Total	7	1	1	1	2	2

In total, 5 subtypes of HPV were detected in malignant parotid gland tumors and were mainly HPV 16 and 18. HPV, human papillomavirus.

**Table V:** Distribution of HPV infection by single or multiple subtypes in benign and malignant parotid gland tumor tissues.

Group	n	Single subtype infection, n (%)	Mixed infection, high- and low-risk subtypes, n (%)	Mixed infection, high-risk subtypes, only, n (%)	Mixed infection, low-risk subtypes only, n
Benign tumor	31	16 (51.6)	6 (19.4)	9 (29.0)	0
Malignant tumor	3	0 (0.0)	1 (33.3)	2 (66.7)	0
Total	34	16 (47.1)	7 (20.6)	11 (32.3)	0

HPV, human papillomavirus.
